# Descriptive Characterization of Psycho-Trauma, Psychological Distress, and Post-traumatic Stress Disorder among Children and Adolescent Internally Displaced Persons in Kaduna, Nigeria

**DOI:** 10.3389/fpsyt.2016.00179

**Published:** 2016-10-28

**Authors:** Taiwo Lateef Sheikh, Abdulaziz Mohammed, Edwin Eseigbe, Tosin Adekeye, Folorunsho Tajudeen Nuhu, Modupe Lasisi, Asiya Muhammad, Zainab Titilope Sulaiman, Aishatu Abubakar Abdullateef, Nafisatu Hayyatudeen, Yusuf Akande

**Affiliations:** ^1^Department of Clinical Services, Federal Neuropsychiatric Hospital, Kaduna, Kaduna, Nigeria; ^2^Department of Psychiatry, Ahmadu Bello University, Zaria, Kaduna, Nigeria; ^3^Department of Paediatrics, Ahmadu Bello University, Zaria, Kaduna, Nigeria

**Keywords:** psycho-trauma, psychological distress, PTSD, children and adolescents, internally displaced persons

## Abstract

**Background:**

A postelection violent conflict in Kaduna resulted in 800 deaths and 65,000 displaced people leading to setting up of camp for internally displaced persons (IDPs). We set out to determine the prevalence and pattern of psycho-traumatic stressful life events, psychological distress, and post-traumatic stress disorder (PTSD) among child/adolescents IDPs.

**Methods:**

A descriptive cross-sectional study of 73 child/adolescent IDPs were selected by total sampling. Stressful life event checklist measured conflict-related trauma and reaction of adolescents to traumatic stress (RATS) measured post-trauma reaction of children/adolescents. Hopkins Symptoms Checklist 37 for Adolescents measured psychological distress associated with trauma. Diagnostic Interview Schedule for Children was used for diagnosis of PTSD.

**Results:**

Of 73 respondents, 3 (4.1%) had probable PTSD, 2 (2.7%) had definitive PTSD, and mean score of the child/adolescent IDPs on HSCL-37A and RATS was 44.7 (SD = 6.3) and 31.9 (SD = 5.7), respectively. Most frequently occurring psychological distress among female participants was suddenly scared for no reason 7 (19.5%) and becoming angry easily and feeling fearful 5 (13.9%). The average score for female participants were higher than that for males on depressive and anxiety subscale of HSCL-37A.

**Conclusion:**

We concluded that children/adolescent IDPs were exposed to psycho-trauma following postelection violent conflict and developed psychological distress. However, the low prevalence of psychological distress and PTSD suggested that living with parents and psychosocial intervention provided could have led to much lower morbidity.

## Introduction

A postelection violent conflict in Northern Nigeria resulted in 800 deaths and 65,000 displaced people over a period of 3 days in April 2011 (1). The majority of the fatalities (88%) were from Kaduna state of Nigeria, known for ethno-religious conflicts. A camp for the internally displaced persons (IDPs) was set up in Kaduna city, the capital of the state to cater for the IDPs. Studies conducted among adults IDPs in the same camp indicated 42% of the 258 adults IDPs had a diagnosis of post-traumatic stress disorder (PTSD) with more than half experiencing 11–15 traumatic events (2). About 60 and 16% of the adult IDPs had probable depression and definite depression, respectively (3). Due to several factors such as developmental stage, level of cognitive and emotional maturity, and limited coping strategies, the psychological reactions in children may differ from those in adults (4). Moreover, studies have shown that stakeholders often underestimate both the intensity and duration of the stress reactions in children (5, 6). Acute stress reactions, adjustment disorder, depression, panic disorder, PTSD, anxiety disorders specific to childhood, and phobias are among mental health morbidities that have been commonly documented following disasters in children (7, 8). In a study of adolescent and youth among displaced Ethiopians, more than 90% of the sample lost their property, 70% suffered extreme thirst, and more than a third witnessed death (9). We set out to determine the prevalence and pattern of psycho-traumatic stressful life events, psychological distress, and PTSD among the children/adolescent IDPs in the Hajj camp in Kaduna, Nigeria.

## Materials and Methods

### Study Setting

The study was conducted in an IDP camp located in Kaduna city, the capital of Kaduna state in the Northwestern zone of Nigeria in April 2013. The camp is located on outskirt of the city in a transit camp originally built for Hajj pilgrims. Over 2,500 IDPs were settled in the camp.

### Study Design

We conducted a cross-sectional study of children and adolescent IDPs aged between 11 and 18 years. The children/adolescents must have been living within the camp and displaced as a result of the violent conflict following the April 2011 elections in Nigeria. We excluded persons already diagnosed with a mental disorder prior to the postelection conflict and those who refused consent either personally or through their parent/guardian.

### Sample Size Determination/Sampling Technique

We did total sampling of all the children in the camp who fulfilled our inclusion and exclusion criteria. The list of the children was gotten from the Social Welfare Committee of the camp. A total of 73 children were interviewed.

### Study Instrument

We designed a sociodemographic questionnaire to measure the sociodemographic characteristics of the child and adolescent IDPs. We used the stressful life event checklist to measure conflict-related trauma. The checklist includes direct experience of physical psycho-trauma and witnessing of others’ trauma. It can be used to assess for the criteria A1 (experienced a traumatic event) in the DSM-IV (10) for the diagnosis of PTSD among adolescents (11). Respondents were to indicate “Yes” or “No” depending on their experience during the conflict. To measure post-trauma reaction of the children and adolescents, we used the reaction of adolescents to traumatic stress (RATS) instrument. This instrument is designed to measure post-traumatic stress as defined in DSM IV (10, 12). The RATS originally a self-administered questionnaire was converted to interviewer administered due to the low level of literacy in the camp. The instrument has 22 questions on the 3 main symptoms of PTSD (hyper arousal, intrusion, and avoidance/numbing), which are all measured on a four-point Likert scale. We defined probable PTSD as any person scoring more than or equal to the 50th percentile score on the RATS questionnaire. The RATS has high structural, content, and construct validity (Cronbach’s alpha for the total score = 0.88 and test–retest reliability stability coefficient = 0.61) (13). We used the Hopkins Symptoms Checklist 37 for Adolescents (HSCL-37A) to measure psychological distress associated with trauma in the participants. The HSCL-37A is a modification of the well-known HSCL-25 and assesses symptoms of internalizing and externalizing problems that have been associated with reactions to trauma (14). It is measured on a four-point Likert scale and scored from 37 to 148. All participants scoring more than the 25th percentile score on HSCL were reinterviewed using the computerized diagnostic interview schedule for children (C-DISC), which was used to make a diagnosis of definitive PTSD. C-DISC (15) is a computerized user-friendly version of diagnostic interview schedule for children (DISC) that can be used to generate both DSM IV and ICD 10 child and adolescent diagnoses. It has been widely used in Nigeria (16). The RATS and HSCL were translated to Hausa, the main language spoken in northern Nigeria, and back translated to English.

### Data Collection and Procedure

We recruited six data collectors who could speak both English and Hausa language fluently and had experience working with and collecting data from IDP camps. They were trained for 5 days on the use of the study questionnaire and interview techniques prior to the onset of the study. Data collection took place over a period of 10 days and the duration of interview ranged from 30 to 50 min. Five supervisors made up of psychiatrists and senior resident doctors supervised the data collectors.

### Data Entry and Analysis

Data were entered into Epi info 3.3.2, cleaned, and edited for inconsistencies before descriptive analysis. The total sampling method used for selection of respondents did not allow for statistical testing of associations.

### Ethical Consideration

Ethical approval was granted by the ethical committee of the Federal Neuropsychiatric Hospital Kaduna before onset of the study. Participating child and adolescents IDPs were required to sign an informed consent form before inclusion in the study. For respondents below 18 years of age, parental consent was also requested. The consent form described the type and purpose of study, and the subject’s rights as a participant in the study, including the right to confidentiality and the right to withdraw from the study. Names were not included in any of the findings, and no financial inducement was given to any respondent. Two psychiatrists, two psychiatric nurses, and a pharmacist provided medical advice if needed, and at the end of the study those determined to have significant distress were referred to the child and adolescent mental health unit of a psychiatric hospital for management.

## Results

### Sociodemographic Characteristics

A total of 73 interviews were conducted. The median age of the children/adolescent IDPs was 13 years (25th and 75th percentile of 12 and 16, respectively). Of the 73 participants, 37 (50.7%) were females, all practiced Islam and were single, 44 (60.3%) were living with both parents, and 57 (78.1%) had both parents alive (Table 1).

**Table 1:** **Sociodemographic characteristics of child and adolescent, IDPs, Kaduna, Nigeria (*n* = 73)**.

Variable	Frequency	Percent
**Gender**		
Male	37	50.7
Female	36	49.3
**Ethnicity**		
Hausa	70	95.9
Others	3	4.1
**Religion**		
Muslim	73	100
**Marital status**		
Single	73	100
**Parent employment (before crisis)**		
Employed	8	11.0
Self employed	64	87.7
Unemployed	1	1.4
**Parent employment (after crisis)**		
Employed	7	9.6
Self employed	38	52.1
Unemployed	28	38.4
**Educational status**		
No formal education	21	28.8
Islamic	6	8.2
Primary	35	47.9
**Caregiver**		
Both parents	44	60.3
One parent	21	28.8
Relative	8	11.0
**Number of parents alive**		
Both	57	78.1
Father	2	2.7
Mother	14	19.0

### Psycho-Traumatic Events on SLE

In the stressful life events concerning family section, the most frequently occurring psycho-traumatic events among female participants were death of a loved one (72.2%) and drastic family changes (33.3%). Among the males, 28 (75.7%) and 15 (40.5%) participants experienced death of a loved one and separation from family against will, respectively (Table 2). In the section on experiencing illness, accidents or disaster, the most frequently occurring psycho-trauma among the females was having experienced an armed conflict (88.9%) and life-threatening medical problems (58.3%). For the male participants, it was having experienced an armed conflict (91.9%) and life-threatening medical problems (67.6%). For both female and male participants, experiencing someone else been mistreated (58.3 and 36.1%, respectively) and been physically assaulted (36.1 and 56.8%, respectively) were the most frequently occurring physical and sexual occurring mistreatment. No female and only 5 (16.1%) male participant reported sexual insult/harassment. Majority of the female 20 (55.6%) and male participants 23 (62.2%) had experienced 5 to 8 of the 12 psycho-traumatic stressful life events.

**Table 2:** **Frequency of stressful life events by gender among child and adolescent IDPs, Kaduna, Nigeria**.

Variable	Female (*n* = 36)		Male (*n* = 37)	
	Frequency	Percent	Frequency	Percent
**Stressful life events concerning the family**				
Death of a loved one	26	72.2	28	75.7
Drastic family changes	12	33.3	14	37.8
Separation from family against will	10	27.8	15	40.5
**Experiences with illness, accidents, and disasters**				
Experience an arm conflict	32	88.9	34	91.9
Life-threatening medical problem	21	58.3	25	67.6
Involvement in a disaster	11	30.6	8	21.6
Involvement in a serious accident	3	8.3	8	21.6
**Physical and sexual mistreatment**				
Seen some one else physically mistreated	21	58.3	30	81.1
Physically assaulted	13	36.1	21	56.8
Seen someone else in great danger	8	22.9	11	29.7
Experience very stressful events of great danger	7	29.0	10	27.0
Sexual insult/harassment	0	0.0	5	16.1
**Number of traumatic events (*n* = 12)**				
1–4	16	44.4	10	27.0
5–8	20	55.6	23	62.2
9–12	0	0.0	5	13.5

### Psychological Distress

The mean score of the children/adolescent IDPs on the HSCL-37A and RATS were 44.7 (SD = 6.3) and 31.9 (SD = 5.7), respectively. The most frequently occurring psychological distress among female participants using HSCL-37A was suddenly scared for no reason 7 (19.5%) and becoming angry easily and feeling fearful 5 (13.9%). Among the male participants, the most frequent psychological distress was loss of interest in opposite sex 6 (16.2%) and someone trying to touch private part/forced to have sex 5 (13.5). The average score and SD of female participants 34.3 (5.2) and 15.1 (3.1) were higher than that for males 29.6 (3.3) and 14.4 (2.2) for depressive and anxiety subscale of HSCL-37A, respectively (Table 3). Of the 73 participants, 22 (30.1%) scored above the 25th percentile and were interviewed with C-DISC.

**Table 3:** **Frequency of psychological distress among children/adolescent IDPs by gender – Kaduna, Nigeria**.

Variable	Female (*n* = 36)		Male (*n* = 37)	
	Frequency	Percent	Frequency	Percent
Suddenly scared for no reason	7	19.5	1	2.7
Becoming angry easily	5	13.9	3	8.1
Feeling fearful	5	13.9	1	2.7
Headaches	4	11.1	2	5.4
Crying all the time	4	11.1	2	5.4
Heart pounding or racing	4	11.1	3	8.1
Feeling restless, can’t sit still	3	8.3	3	8.1
Difficulty falling asleep, staying asleep	3	8.3	2	5.4
Worrying too much about things	2	5.6	2	5.4
Poor appetite	2	5.6	2	5.4
Feeling blue	2	5.6	1	2.7
Feeling no interest in things	2	5.6	1	2.7
Feeling of being trapped or caught	2	5.6	0	0
Feeling everything is an effort	1	2.8	2	5.4
Trembling	1	2.8	1	2.7
Faintness, dizziness or weakness	1	2.8	1	2.7
Feeling tensed or keyed up	1	2.8	0	0
Feeling hopeless about the future	1	2.8	0	0
Feeling low in energy, slowed down	1	2.8	0	0
Drinking alcohol when I go out at weekends	1	2.8	0	0
Loss of interest in opposite sex	1	2.8	6	16.2
Has someone tried to touch your private parts against your will or forced you to have sex	0	0	5	13.5
Feeling lonely	0	0	1	2.7
Starting fights	0	0	1	2.7
Intentionally hurting someone	0	0	1	2.7
Spells of terror or panic	0	0	1	2.7

**Subscale of HSCL**	**Mean**	**SD**	**Mean**	**SD**

Depression scale	34.3	5.2	29.6	3.3
Anxiety scale	15.1	3.1	14.4	2.2

### Probable and Definitive PTSD

Out of the 73 children/adolescent IDPs interviewed, only 3 (4.1%) female participants had probable PTSD (scored more than or equal to the 50th percentile score of 44 on RATS). Of the 22 participants who scored above the 25th percentile on HSCL-37A, 2 were diagnosed as having definitive PTSD using C-DISC. The prevalence of PTSD among the participants, therefore, was 2.7%. The female participants had higher average score than the male participants in the intrusion (10.8 vs. 9.6), avoidance (13.6 vs. 11.8), and hyperarousal (9.1 vs. 8.9) subscales of RATS (Table 4).

**Table 4:** **Frequency of reactions of child/adolescent IDPs to psycho-trauma – Kaduna, Nigeria**.

Variable	Female		Male	
	Frequency	Percent	Frequency	Percent
**Intrusion symptoms**				
Think often of event(s) even if do not want to	17	47.3	9	24.3
Feel afraid or sad if thinking about event(s)	14	38.9	9	24.3
Thinking about event(s) have strong feelings in body	10	27.7	3	8.1
Have bad dreams or nightmare about the event(s)	5	13.9	3	8.1
Have feeling event is happening all over again	1	2.8	3	8.1
Find self sometimes acting as time of event(s)	1	2.8	2	5.4

**Total average score**	**Mean**	**SD**	**Mean**	**SD**

	10.83	0.8459	9.62	0.7342
**Avoidance/numbing**				
Try to stay away from people, places, or things that remind of event(s)	10	27.7	2	5.4
Try to push away feelings about event	7	19.5	6	16.2
Try not to think or talk about event(s)	7	19.5	0	0
Have trouble expressing feelings	4	11.1	2	5.4
Forgotten important things about event	3	8.3	2	5.4
Don’t think positively about future	1	2.8	1	2.7
Feel all alone	1	2.8	0	0
Do not feel close to people around	1	2.8	0	0
Feel uninterested in things like sports, friends, school, and family	0	0	0	0

**Total average score**	**Mean**	**SD**	**Mean**	**SD**

	13.58	0.7318	11.76	0.5756
**Hyperarousal**				
Startle easily when you hear a loud sound or when something surprises you	9	25.0	4	10.8
Have trouble concentrating or paying attention	2	5.6	1	2.8
Alert (always watching out or on guard)	1	2.8	5	13.5
Have trouble falling asleep	1	2.8	1	2.7
Have trouble staying asleep/wake up too early	1	2.8	1	2.7
Often have arguments with others	0	0	2	5.4

**Total average score**	**Mean**	**SD**	**Mean**	**SD**

	9.14	0.5697	8.86	0.6056

## Discussion

This study investigated prevalence and pattern of psycho-trauma, psychological distress, and PTSD among children/adolescents in IDP camp following postelection violent conflict in Northern Nigeria. In the study, the most frequently occurring psycho-traumatic events among children/adolescent IDPs were death of a loved one and experiencing armed conflict. Majority had experienced five to eight psycho-traumatic stressful life events. The most frequent psychological distress among female participants was suddenly scared for no reason and becoming angry easily, while loss of interest in opposite sex and someone trying to touch private part or been forced to have sex among males. Three female participants had probable PTSD, while two had definitive PTSD. The female participant consistently had higher score on the three subscales of RATS.

Majority of the children/adolescent IDPs had experienced five to eight traumatic events. This is lower than 11–15 trauma events exposure of majority of adult population studied from the same IDPs camp in an earlier study (2). This will suggest that the children/adolescents IDPs had less exposure to trauma compared with the adult population but the findings may be due to experience of destruction of “destruction of personal properties” by adults, which children/adolescent IDPs may not report. The most frequent psycho-trauma among the children/adolescent IDPs was death of a loved one and experiencing armed conflict. This is similar to the report of Bean et al. in a study comparing psychological distress in unaccompanied refugee minors (11). Male children/adolescent IDPs consistently had experienced more traumatic events in all the three domains of the stressful life event questionnaire (stressful life events concerning the family, experiences with illness, accidents and disasters, and physical and sexual mistreatment) with the exception of experiencing sexual insult/harassment and very stressful events of great danger. Similar pattern was also observed in the adult IDPs study (2).

The mean score of the children/adolescent IDPs on the HSCL-37A and RATS scales were 44.7 and 31.9, respectively, in this study. Two previous studies with larger sample size than this study found mean scores of 56.9 and 63.1 for HSCL and 39.3 and 41.4 for RATS (14, 17). This suggests the children and adolescent IDPs in this study had less psychological distress than those of the two previous studies. In this study, two of the three female adolescent IDPs screened by RATS to have probable PTSD also had definitive PTSD independently assessed by C-DISC, given a prevalence of probable PTSD and definitive PTSD of 4.1 and 2.7%, respectively. The lower prevalence for definitive PTSD is understandable due to the use of more specific diagnostic tool (C-DISC) compared with the less specific RATS used to make a diagnosis of probable PTSD. The prevalence of either probable or definitive PTSD found in this study is very low when compared with a pool prevalence of 47% from a meta-analysis of studies of mental health in children and adolescents living in areas of armed conflict (18). The overall lower prevalence of psychological distress and PTSD may partly be explained by the 2 years difference between the conflict and the study, but evidence from a 2-year follow-up study suggested that psychological distress/PTSD among children and adolescent exposed to trauma do not change significantly over time especially if they are without care giver or protection (19). Unaccompanied children and adolescent exposed to psycho-trauma have been shown to have higher psycho-traumatic experiences and subsequent psychological distress and morbidity than those accompanied by their parents or guardian (20). In this study as evident by the sociodemographic information of the children/adolescents, they were mostly living with their parents in a secured IDPs camp. The camp was assisted in setting up medical, psychological, and social support for the adult and children IDPs through the psychosocial intervention frame work of a Nigerian Federal Government owned Neuropsychiatric Hospital in the locality (21). A secured living environment for the children and adolescent IDPs while accompanied by parents or care givers and a proactive psychosocial intervention by a stakeholder may be a more likely explanation for the lower than expected psychological distress among the participants.

The mean score for psychological distress for both depression and anxiety subscales was higher for females compared with male adolescent IDPs. This result is consistent with the findings of higher rates of depression and anxiety among Burmese adolescents students exposed to psycho-trauma in Thailand (14). This is despite male participants having been exposed to more psycho-trauma overall compared with females. Although studies have consistently found higher rates of depression and anxiety among females compared with males (22), other factors may have contributed to this finding. The HSCL used for this study may have picked more distress in females solely because females were more likely to express distressing feelings than males (23). Reports of sexual harassments and been forced to have sex were by male children/adolescent IDPs; this is similar to the findings among traumatized Burmese adolescent where males reported more sexual harassment/abuse (14). This finding could be an under reporting or total lack of reporting (as in this case) of sexual assault by females due to the culturally sensitive nature of disclosing sexual harassment in Northern Nigeria. Although female adolescent IDPs were less exposed to psycho-trauma, all of the probable and definitive PTSD cases were females. This finding is similar to that of the adults IDPs (3) and could be explained by the higher risk of developing PTSD by females (24).

The findings of this study are subjected to the following limitations. The study used total sampling, and therefore there was no power calculation to determine appropriate sample size for test of significant association. Therefore, no statistical test of association was conducted. The sample size of 73 could be considered to be small, and therefore, there should be caution in generalizing the findings of this study beyond the population studied. We did not quantitatively evaluate the role of the psychosocial intervention and parental social support offered to the children and adolescent IDPs.

## Conclusion

We concluded that children and adolescent IDPs living in Hajj camp in Kaduna, Nigeria were exposed to psycho-trauma following postelection violent conflict and developed psychological distress. However, the low prevalence of the psychological distress and PTSD suggested that psychosocial intervention could have led to much lower morbidity.

## Author Contributions

TS: conceptualisation, design, analysis, and overall supervision of the entire research work. AM: design, analysis, supervision of data collection, result presentation, and writing of manuscript. EE: design, data collection, and analysis. TA: administration of psychometric instruments, data collection, and manuscript writing. FN: supervision of data collection, administration of instruments, and analysis. ML: data collection and administration of instruments. AM: literature review, data collection, administration of instruments, and analysis. ZS: literature review, data collection. AA: analysis. NH: data collection. YA: literature review, design, and statistical analysis.

## Conflict of Interest Statement

The authors declare that the research was conducted in the absence of any commercial or financial relationships that could be construed as a potential conflict of interest.
